# Highly efficient removal of lead and cadmium during wastewater irrigation using a polyethylenimine-grafted gelatin sponge

**DOI:** 10.1038/srep33573

**Published:** 2016-09-16

**Authors:** Bingbing Li, Feng Zhou, Kai Huang, Yipei Wang, Surong Mei, Yikai Zhou, Tao Jing

**Affiliations:** 1 State Key Laboratory of Environment Health (Incubation), Key Laboratory of Environment and Health, Ministry of Education, Key Laboratory of Environment and Health (Wuhan), Ministry of Environmental Protection, School of Public Health, Tongji Medical College, Huazhong University of Science and Technology, Wuhan, Hubei, 430030, China; 2 Institute of Environmental Pollution and Health, School of Environmental and Chemical Engineering, Shanghai University, Shanghai 200444, China

## Abstract

Wastewater irrigation is a very important resource for heavy metal pollution in soil and then accumulation in vegetable crops. In this study, a polyethylenimine (PEI)-grafted gelatin sponge was prepared to effectively adsorb heavy metals during wastewater irrigation. Based on the strong water adsorption ability, wastewater remained in the PEI-grafted gelatin sponge for a sufficient time for the heavy metals to interact with the sorbents. The binding capacities of Pb(II) ions and Cd(II) ions on the PEI-grafted gelatin sponge were 66 mg g^−1^ and 65 mg g^−1^, which were much more than those on the gelatin sponge (9.75 mg g^−1^ and 9.35 mg g^−1^). Subsequently, the PEI-grafted gelatin sponge was spread on the surface of soil planted with garlic and then sprayed with synthetic wastewater. The concentrations of cadmium and lead in the garlic leaves were 1.59 mg kg^−1^ and 5.69 mg kg^−1^, respectively, which were much lower than those (15.78 mg kg^−1^ and 27.98 mg kg^−1^) without the gelatin sponge, and the removal efficiencies were 89.9% and 79.7%. The PEI-grafting gelatin sponge could effectively remove heavy metals during wastewater irrigation, which improved the soil environment and reduced human exposure to heavy metals.

Water shortages in the world have led to the reuse of municipal wastewater for irrigation. In many developing countries, wastewater irrigation is a very important way in agricultural production, however, it would increase human exposure to the pollutants. Amin *et al.* found that vegetables grown in the Mardan District of Pakistan were highly polluted by heavy metals due to the irrigation of untreated wastewater, which would lead to serious health risks for local people^1^. Jiang *et al.* reported that agricultural soil was potentially at risk of heavy metal accumulation from irrigation water, which was higher than those from atmospheric deposition and fertilizer^2^. Thus, decrease the contaminants in wastewater system was an important way to avoid their entrance into agricultural soil and then food chain.

Various technologies were proposed to decrease the heavy metal concentration in wastewater, such as ion exchange, adsorption, chemical precipitation and membrane separation^3^,^4^,^5^,^6^,^7^. Among them, adsorption was considered as a flexible, convenient and low cost technology. After simple desorption, the adsorbents are regenerated for multiple uses and it still has a high efficiency^4^,^8^. Therefore, it was a preferred process for wastewater pollution control. However, developing a specific sorbent with high adsorption capacity is an interesting yet still challenging task. Currently, carbon materials (such as carbon foam, carbon nanotubes, graphene and activated carbon)^9^,^10^,^11^, nanosized metal oxides (such as ferric oxides, manganese oxides and zinc oxides)^4^, chitosan composites^12^,^13^ and low-cost adsorbents (such as zeolites and weathered coal)^14^,^15^,^16^ are all considered in the literatures to be promising choices for the heavy metal pollution control. Unfortunately, it is impossible to recover these materials and they are often used in a sewage station based on the special operating devices. Wastewater irrigation is different from wastewater emission, which is an unpalatable choice in water scarce countries. They weren’t even sure about the sources of irrigated wastewater and dry season. Due to the economic costs, it is difficult for farmers to develop a purification system by themselves to further decrease the amounts of heavy metals using these materials as the sorbents.

The easiest and most direct way is to spread a cheap material on the soil surface to remove heavy metals during wastewater irrigation. In other words, this technique implies that the following two conditions are necessary: this material has high adsorption capacities for the contaminants and allows wastewater to flow through at a slow flowing rate to provide sufficient time for the removal of heavy metals. A gelatin sponge is an easily available biopolymer that not only has good chemical, physical, and biological stability but also contains different types of functional groups, which can interact with heavy metals by different forces^7^,^17^,^18^. Most importantly, although the water absorption of a gelatin sponge is very high, the volume does not noticeably increase and a highly porous structure is still maintained for the fast adsorption kinetics^19^.

However, due to its crosslink with the amino groups, adsorption capacity of a gelatin sponge is not very high. Surface modification can be used to further improve its adsorption capacity and mechanical stability. Branched poly(ethyleneimine) (PEI) is a water soluble polymer and the amine groups exist in primary, secondary and tertiary forms in any given chain segment^20^,^21^,^22^. Thus, it can be used for heavy metals adsorption in real water samples due to the formation of chelation complexes^23^. In this study, a PEI-grafted gelatin sponge was prepared and spread on the surface of soil planted with garlic to decrease the heavy metal concentrations during wastewater irrigation, because of its following properties: (1) the PEI-grafted gelatin sponge with a high adsorption capacity could be used for the heavy metal pollution control; (2) the high water adsorption ability can provide sufficient time to decease the amounts of heavy metals in wastewater.

## Results and Discussion

### Synthetization and characterizations of PEI-grafted gelatin sponge

**Figure 1:**
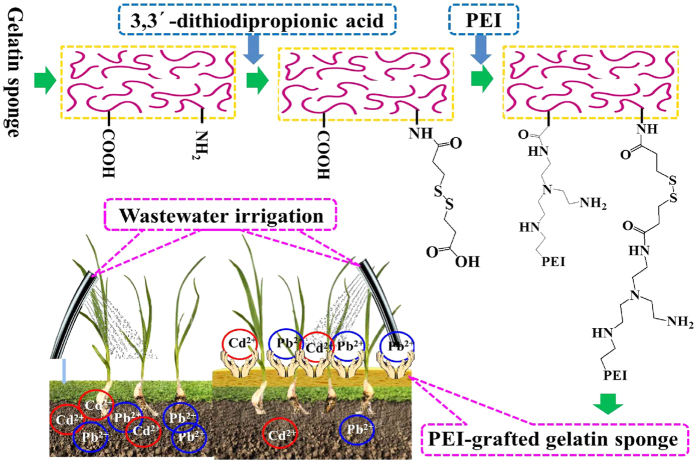
Preparation of the PEI-grafted gelatin sponge and its application in the removal of heavy metals during wastewater irrigation.

In this study, preparation of PEI-grafted gelatin sponge and its application are shown in Fig. 1. Due to the introduction of 3,3′-dithiodipropionic acid, the amino groups of gelatin sponge were converted to the carboxylic groups by the formation of amide bonding between the carboxylic acid groups of the 3,3′-dithiodipropionic acid and the amino groups of gelatin. Then, the target product PEI-grafted gelatin sponge was further prepared based on the ionic interactions between the amine groups of PEI and the carboxylic groups (3,3′-dithiodipropionic acid and gelatin) based on the NHS/EDC coupling. The number of amino groups increased significantly and the mechanical stability was improved based on the modification of PEI.

Supplementary Fig. S1 indicates the SEM micrographs of the gelatin sponge and PEI modified-gelatin sponge. The surface morphologies were considerably different. The porous three-dimensional (3D) structure of the gelatin sponge was a fibrous network, whereas a lamellar membrane was observed on the PEI modified-gelatin sponge. Zeta potential measurement was used to study the modified mechanism. It was shown that the potential of the gelatin sponge was +12.14 mV in aqueous solution (pH = 5.0), since the gelatin had a net positive charge on the surface at low pH. But, after modified by the 3,3′-dithiodipropionic acid, it changed to −13.78 mV, because of the presence of carboxyl groups. Finally, this value of PEI modified-gelatin sponge was +27.43 mV, implying a PEI layer was created around gelatin sponge.

FT-IR spectra were obtained to verify the change of function groups on the gelatin sponge (see Supplementary Fig. S2). The infrared spectrum of the gelatin sponge exhibited broad bands assigned to O-H and N-H stretching centered at 3429 cm^−1^. The characteristic peak of C = O and C-N at 1629 cm^−1^ and N-H stretching at 1537 cm^−1^ were attributed to amide I and amide II. The peak emerging at 1336 cm^−1^ implied the presence of amide III^24^. After modification with PEI, the band intensity peak at 3429 cm^−1^ was greatly increased and the peaks of the peptidic bond of the protein were not changed, which indicated that the enhancement of amino groups resulted from PEI.

The surface chemical compositions of the gelatin sponge and PEI-grafted gelatin sponge were studied based on the XPS spectra. It is shown that the nitrogen atom concentration was increased from 11.91% to 17.35%, implying the successful modification of PEI on the gelatin sponge. For the gelatin sponge, the N1s peak at 399.75 eV appeared due to the NH (NH_2_) groups, and the O1s peak at 531.7 eV attributed to the C-OH/C-O-C groups (see Supplementary Fig. S3)^24^,^25^. After PEI modification, a new S1s peak at 167.4 eV was assigned to the -S-S- groups due to the introduction of 3,3′-dithiodipropionic acid.

### Effect of pH

The pH value was an important parameter for the removal efficiency of the sorbents, because it not only changed the form of heavy metals, but also had a significant influence on the species of functional groups. As shown in Supplementary Fig. S4, the adsorption capacities increased with the increasing of pH value and reached a plateau when this value changed from 4.0 to 7.0. Subsequently, the adsorption capacities were significantly decreased in basic solution because the concentration of OH^−^ increased and the lead ions or cadmium ions might exist in the forms of X(OH)^+^ and X(OH)_2_ (X = Pb or Cd)^26^,^27^. Thus, the percentage removal of Cd^2+^ (or Pb^2+^) by precipitation was much greater than that by adsorption at a pH of above 7.0. From the perspective of electrostatic interaction, at low pH values, hydrogen ions could compete with target ions for the active sites, resulting in the decrease of binding capacities. When the pH value was increased, competition from hydrogen ions and the electrical repulsion force were all decreased, and the removal efficiency was gradually increased^28^. Thus, the proposed sorbents can be used for the adsorption of heavy metals in surface water or wastewater due to the formation of chelation complexes^28^. Furthermore, PEI could be used as a proton acceptor for the carboxyl group of gelatin, which would facilitate the binding of the carboxylate to metal ions. Thus, a pH of 6.0 was fixed to remove lead ions and cadmium ions from the real water samples.

### Adsorption for lead and cadmium

**Figure 2:**
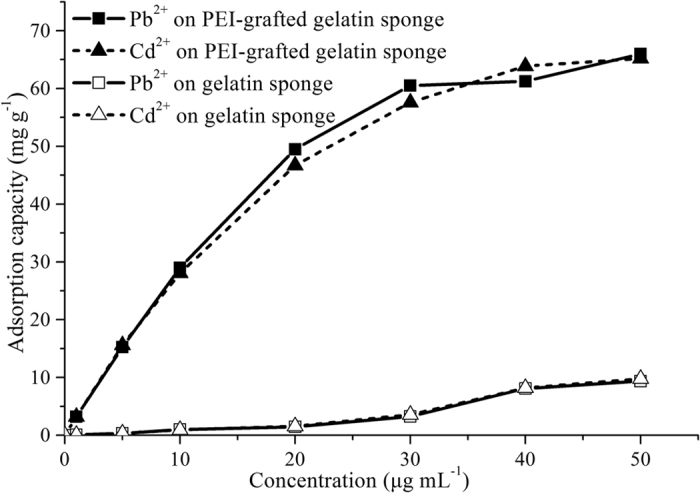
Adsorption isotherms of heavy metals on the gelatin sponge and the PEI-grafted gelatin sponge.

The PEI-grafted gelatin sponge was applied as a sorbent to adsorb heavy metals in wastewater and a common gelatin sponge was also studied for comparison. The evaluated methods are shown in the Supporting Information. The adsorption capacities of lead ions and cadmium ions on the PEI-grafted gelatin sponge were 66.1 mg g^−1^ and 65.2 mg g^−1^, respectively, which were more than those of the gelatin sponge (9.35 mg g^−1^ and 9.75 mg g^−1^) (Fig. 2). Gelatin as a biological macromolecule had different types of functional groups, such as -COOH, -NH_2_, -OH and -SH, which could be applied to remove target ions (see Supplementary Fig. S5). The amino groups could be used not only to remove the heavy metals in wastewater due to the formation of chelation complexes, but also as a proton acceptor for the carboxyl group of gelatin, which would facilitate the binding of the carboxylate to metal ions. Furthermore, the gelatin could also form complexes with metal ions through various side groups such as imidazole and thiol. Further modification of PEI could significantly increase the number of amino groups to enhance the removal efficiency of sorbents.

The Freundlich isotherm and Langmuir isotherm are often used to study the adsorption mechanism of the PEI-grafted gelatin sponge. In this study, the adsorption parameters are shown in Table 1. The adsorption capacities of Pb(II) ions and Cd(II) ions onto the PEI-grafted gelatin sponge were increased with the increasing of original concentration, and the experimental data was good fit with the Langmuir model. It was shown that the removing mechanism was the controlled monolayer adsorption. Based on the Langmuir model, the Q_max_ values of the PEI-grafted gelatin sponge were 80.6 mg g^−1^ for lead ions and 79.9 mg g^−1^ for cadmium ions, respectively. Dimensionless constant named as equilibrium parameter was applied to indicate the essential characteristics of adsorption model (Supplementary R_L_). These values for both of the test ions on the PEI-grafted gelatin sponge were 0.24, which demonstrated that the experimental parameters were favorable for the removal of heavy metals.

### Adsorption kinetic studies for Pb(II) ions and Cd(II) ions

The adsorption kinetics of the PEI-grafted gelatin sponge for the target ions was studied in this study, especially in wastewater irrigation. Due to the water transmission characteristics of soil, the adsorption of heavy metal ions must be completed when the wastewater flows from the surface of the sponge to the bottom layer. Otherwise, it would lead to soils polluted by heavy metals and toxicological effects for vegetable crops. The high water adsorption ability of PEI-grafted gelatin sponge could slow the transit of wastewater to the soil and provide sufficient time for the adsorption of target pollutants on the sponge. Unlike general removing procedure at a fixed flow rate, remain of wastewater on the sponge was a complex kinetics procedure and there were a complex interaction among water molecules, PEI-grafted gelatin sponge, heavy metal ions and soil. Therefore, shaking adsorption procedure was performed to investigate the interaction between the sponge and heavy metals and water losing experiment was used to evaluate the kinetics procedure of water.

**Figure 3:**
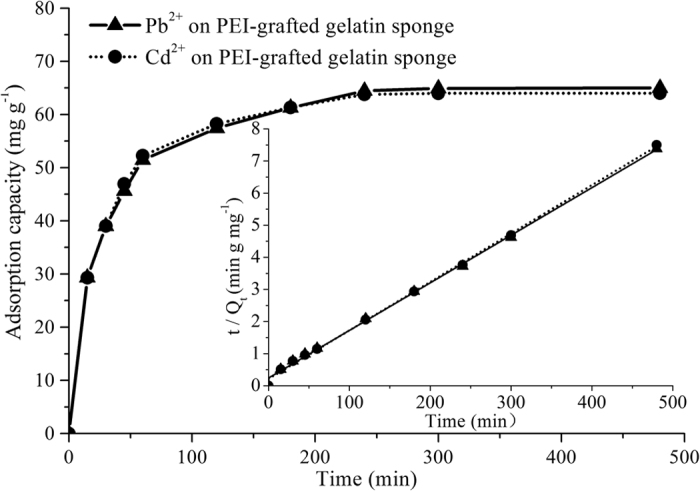
Rebinding kinetic behavior of of heavy metals on the PEI-grafted gelatin sponge. Inset: the pseudo-second-order mode for the adsorption of heavy metals.

The results of the shaking adsorption experiments indicated that more than 54.7% of the total metals were remained on the PEI-grafted gelatin sponge in the first 15 minutes (Fig. 3), which demonstrated that the removal rate was quite rapid, which was beneficial to decrease heavy metal pollution during wastewater irrigation. The results of the water losing experiments indicated that the outflow of water on the wet PEI-grafted gelatin sponge was changed considerably within the first 30 minutes and then gradually slowed over time. The results indicated that 45.5% water still remained on the sponge after 15 min (Fig. 3). Furthermore, the water molecules organized around the PEI-grafted gelatin sponge could be competitively adsorbed by sodium chloride (NaCl) and then induced a salting-out effect. The curve of the remaining water was not changed even if the concentration of NaCl was 0.1 g mL^−1^, which implied that the PEI-grafted gelatin sponge had high water adsorption ability.

**Figure 4:**
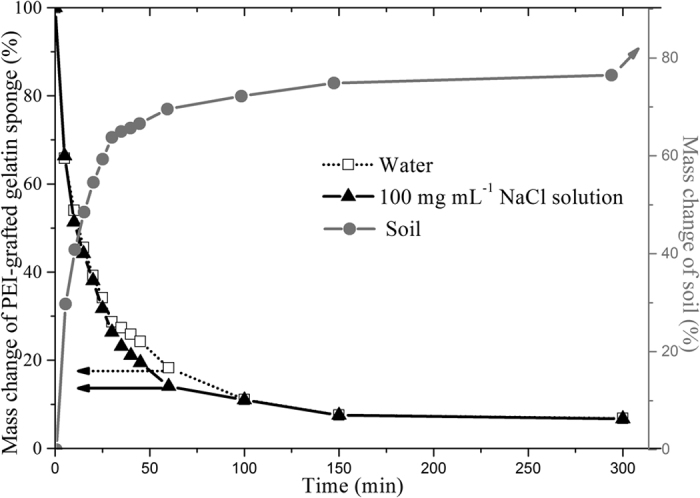
Retention behavior of water on the PEI-grafted sponge and the effect of sodium chloride on the retention behavior.

To further study the mechanism of adsorption kinetics, intra-particle diffusion, pseudo-first-order and pseudo-second-order were performed to investigate the adsorption dynamics of heavy metals on the PEI-grafted gelatin sponge. The kinetic equations are given in the Supporting Information. It was shown that high correlation coefficients (R^2^) were obtained, and the calculated values of the binding capacity (Q_e,calc_) were the same as the experimental values (Q_e,exp_) (Fig. 4, Table 2). Therefore, the adsorption kinetic procedure of heavy metals on the PEI-grafted gelatin sponge was the pseudo-second-order kinetic procedure. Furthermore, the intra-particle diffusion model was further used to describe the adsorption process of metal ions. The plots exhibited double linearity, implying there were two steps in the adsorption process (see Supplementary Fig. S6).

### Stability and regeneration

The stability of PEI-grafted gelatin sponge was studied by investigate the adsorption capacities of heavy metals after 90 d of storage in aqueous solution. It is shown that removal efficiency was almost unchanged, implying the excellent time stability. Since the adsorption of heavy metals onto the sorbent was a reversible process, a sponge was repeatedly putted into the wastewater samples and taken out for regeneration using an acetic acid solution (0.1 mmol L^−1^). The results showed that the removal ability was decreased with the increasing of usage, over 80% efficiency was received after the fifth adsorption-desorption cycles, implying that the PEI-grafted gelatin sponge was cost-effective. However, performances of the sponge were affected by several factors in the natural environment, such as sunshine, wind and so on. Thus, PEI-grafted gelatin sponge was spread on the soil surface in the Taiyuan city of china for two weeks (Weekly mean of temperature: 17 ± 1.1–31 ± 2.4 °C, Weekly mean of relative humidity: 57%; Rainy Day: 2 days; Precipitation: 11 mm; breeze). Then, the weight and the adsorption capacities were investigated to indicate the degradation of PEI-grafted gelatin sponge in natural condition. It was shown that the loss weight was 6.3 ± 1.2% and the adsorption capacities for lead ions and cadmium ions were 57.8 mg g^−1^ and 58.2 mg g^−1^, which were 87.4% and 89.2% of the original adsorption capacities, respectively. Thus, we recommended that the PEI-grafted gelatin sponge was changed once a week, due to ease of operation and low cost.

### Removal of metal ions during wastewater irrigation

Dietary intake is the main route of heavy metal exposure, especially in vegetable crops irrigated with metal-contaminated wastewater. Excessive uptake of heavy metals could result in serious health problems in humans. Garlic (*Allium sativum* L.) was widely used as food, spice and medicine, which has some beneficial effect in preventing heavy metal induced alterations, but people seldom focus on the accumulation of pollutants^29^.

**Figure 5:**
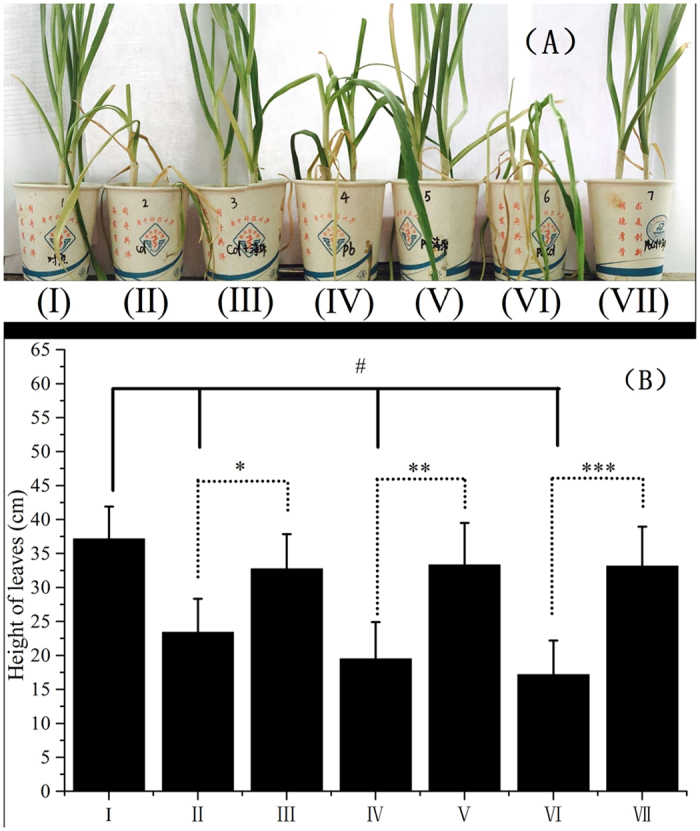
Photograph of garlic one month after treatment (**A**); leaf growth of garlic after one month (**B**). (I) Blank control (spraying with ultrapure water); (II) positive control of Cd(II); (III) treatment group of Cd(II); (IV) positive control of Pb(II); (V) treatment group of Pb(II); (VI) positive control of mixed contamination; and (VII) treatment group of mixed contamination. Notes: *Denotes a significant difference using a T-test. ^#^Denotes a significant difference based on analysis of variance.

Therefore, the PEI-grafted gelatin sponge was spread onto the surface of soil planted with garlic to effectively remove target ions during wastewater irrigation. The average length of garlic leaves decreased significantly in the positive control (23.4 and 19.5 cm) and the mixed contamination group (17.2 cm) as compared to the blank control (37.2 cm) (Fig. 5). Compared with the treatment groups and the blank control, leaf drooping of garlic was found in the positive control and mixed contamination group, which demonstrated a significantly toxic effect on the plant. In addition, the results demonstrated that the average lengths of garlic leaves in the positive control were much lower than those in the treatment groups (P < 0.05). There was no statistically difference of garlic growth among blank control (I), treatment groups (III and V) and mixed treatment groups (VII) (F = 2.27, P > 0.05), implying a high removal efficiency of heavy metals by the PEI-grafted gelatin sponge during wastewater irrigation.

**Figure 6:**
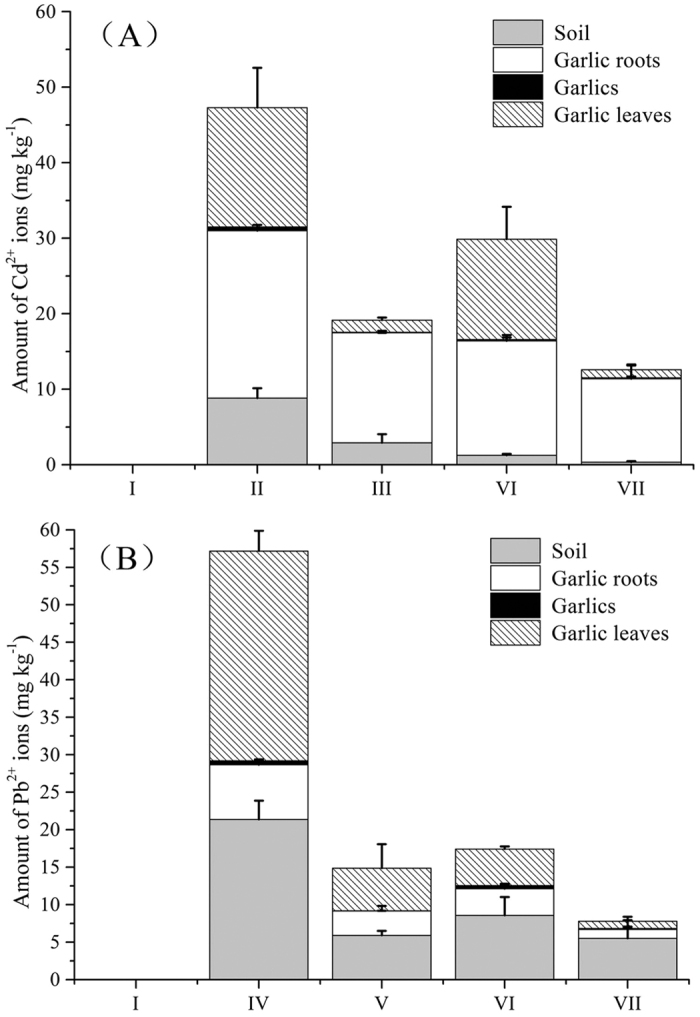
Bioaccumulation of Cd(II) (A) and Pb(II) (B) in the soil and edible parts of vegetable crops with or without the spreading of the PEI-grafted sponge. Notes: (I-VI) denote the same contents as in Fig. 5. Each group has three cups and two garlic plants were planted in each cup. The concentration in the sprayed solution were 500 mg L^−1^ (signal contamination) and 250 mg L^−1^ (mixed contamination), respectively. The sprayed volume was 10 mL day^−1^ and the treatment time was one month.

Figure 6 shows that the concentrations of Cd(II) ions and Pb(II) ions in the soil were 2.91 mg kg^−1^ and 5.92 mg kg^−1^, respectively, which is much lower than those (8.82 mg kg^−1^ and 21.37 mg kg^−1^) without the protection of PEI-grafted gelatin sponge, and the removal efficiencies were 67.0% and 72.3% (Fig. 6). However, there was no difference of Cd(II) content in the roots with and without the protection of the sponge, which should attract sufficient attention for cadmium pollution. For the positive control of Cd(II), 57.7% and 41.1% of Cd(II) was accumulated in the roots and leaves of garlic, respectively. By using the PEI-grafted gelatin sponge, the amount of Cd(II) in the leaves changed from 15.78 to 1.59 mg kg^−1^, and the removal efficiency was 89.9%. When the soil was simultaneously contaminated by Pb(II) and Cd(II), the PEI-grafted gelatin sponge with high adsorption capacity could still effectively remove Cd(II) from the wastewater and then decrease the risk of heavy metals. The removal efficiencies were 71.4% and 92.4% for the soil and leaves, respectively.

For the Pd(II) in the positive control (IV), 19.2% and 73.7% of Pd(II) was accumulated in the root and leaves of garlic, respectively. Most of the Pd(II) ions remained in the soil and also accumulated in the leaves. After using the PEI-grafted gelatin sponge, the amount of Pd(II) in the leaves changed from 27.98 to 5.69 mg kg^−1^ and the removal efficiency was 79.7% (Fig. 6). The amount of Pd(II) in the roots also decreased from 7.3 mg kg^−1^ to 3.23 mg kg^−1^ and the removal efficiency was 55.75%. Similar results were obtained when the soil was simultaneously contaminated by Pb(II) ions and Cd(II) ions.

It was shown that PEI-grafted gelatin sponge could be applied for the pollution control during wastewater irrigation to improve the soil quality and then reduce the exposure of crops to heavy metals. This study provided a simple, effective and economic technology to prevent further contamination of heavy metals in the soil. For the contaminated soil, the removal ability of PEI-grafted gelatin sponge for heavy metals was very weak. The removal efficiency was only 5.3%, when the dry PEI-grafted gelatin sponge was spread onto the surface of wet soil.

## Conclusion

In this study, a PEI-grafted gelatin sponge was prepared and spread onto the surface of soil planted with garlic to decrease the risk of heavy metals during wastewater irrigation. According to the strong water adsorption ability and the high removal efficiency of the sponge for heavy metals, wastewater remained in the PEI-grafted gelatin sponge for a sufficient period of time for the heavy metals to interact with the sorbents. Garlic was used as the test plant to study the removal efficiency of the PEI-grafted gelatin sponge during wastewater irrigation. The removal efficiencies for lead ions and cadmium ions were 89.9% and 79.7%, respectively. Furthermore, the PEI-grafted gelatin sponge had the advantages of excellent mechanical stability, environmental friendliness, ease of operation, low operating costs and high adsorption capacity. We believe that this study provide an effective, practical and direct technology to improve the soil quality and decrease the accumulation of heavy metals.

## Methods

### Materials

Absorbable gelatin sponges (60 mm × 20 mm × 5 mm) were purchased from Nanchang Xiangentang medical equipment Co. Ltd. (Xixiang, China). N-hydroxysuccinimide (NHS), branched polyethylenimine (PEI) with a molecular weight of 800 Da, 3,3′-dithiodipropionic acid, ethanol, dimethylformamide and 1-ethyl-3-(3-dimethylaminopropyl) carbodiimide hydrochloride (EDC) were purchased from Sigma-Aldrich (St. Louis, MO, USA). Stock aqueous solutions of lead and cadmium (1 mg mL^−1^) were purchased from the National Institute of Metrology of China and then dissolved with ultrapure water.

### Preparation of PEI-grafted gelatin sponge

A PEI-grafted gelatin sponge was synthesized according to the literature with a modification^30^. 3,3′-dithiodipropionic acid solution (0.2 mol L^−1^) was prepared by using aqueous dimethylformamide and then mixed with a 0.1 mol L^−1^ of EDC solution. The mixture was stirred at 40 °C for 1 h and then slowly added dropwise into 10 mL of acetic acid solution (pH = 5.0). A piece of absorbable gelatin sponge was introduced into the resulting solution. The reaction was performed at 40 °C for 24 h with continuous shaking (200 rpm) to convert the amino groups to the carboxyl groups. The sponge was removed from solution using a tweezers and placed on a paper to remove the remaining water. Subsequently, 0.5 mmol EDC, 0.5 mmol NHS and 0.5 mmol PEI were dissolved by 20 mL of acetic acid solution (pH = 6.5) and the resulting sponge was introduced to the mixture, which was incubated at 40 °C for 24 h with continuous shaking (200 rpm). The final product was received using a tweezers and washed three times with water.

### Adsorption experiments

The adsorption abilities of the gelatin sponge and PEI-grafted gelatin sponge were investigated with batch shaking adsorption experiments. Approximately 0.2 g of each sponge was placed into 20 mL of aqueous solution (pH = 6.0) spiked with different concentrations of heavy metals (lead and cadmium as the test metal ions). After shaking for 12 h (at 200 rpm), the amounts of lead ions and cadmium ions in the supernatant were detected by an inductively-coupled plasma atomic emission spectroscope (ICP-AES, Perkin Elmer Plasma 3200RL). Kinetic studies of PEI-grafted gelatin sponge were the same as the adsorption procedure, however the testing samples were taken at scheduled time intervals.

### Removal of lead ions and cadmium ions during wastewater irrigation

Garlic growth assays were carried out using an indoor cultivation method (temperature was 25 °C and sunshine hours was 8 h) to verify the application perspective of the PEI-grafted gelatin sponge. Garlic is a bulbous plant and garlic growth is easy. Thus, it was used to investigate the bioaccumulation of pollutants in the fruit, leaves and roots of garlic after spreading with a gelatin sponge. Soils for the incubation studies were sampled from the city park soils in September 2015 (sand 120 g kg^−1^, silt 410 g kg^−1^, and clay 470 g kg^−1^). The soils were then packed to a paper cup (I.D. = 15 cm and the packed height = 6 cm) for the garlic growth.

Fresh garlic bulbs were chosen from the local farmers market and then each clove was pushed into the soil to a depth of approximately 1.5 cm. Subsequently, the PEI-grafted gelatin sponge was spread on the soil surface and surrounded the garlic clove. Lead and cadmium are commonly present in different types of industrial effluents and mainly responsible for environmental pollution and public health. Thus, it was sprayed with lead (or cadmium) solutions (0.5 mg mL^−1^, 50 mL) at a flow rate of 1 mL min^−1^. The positive controls without the sponge spreading and a blank control (spraying with water) were also included in the experiments. After 30 days of growth, all of the plants were harvested (leaf, garlic, and root), washed with tap water to remove the soil, and then dried in an oven (105 °C, 30 min). The concentrations of target ions in the dried plants and soil were determined by ICP-AES after digestion with the mixture of HNO_3_ and HClO_4_ (1/4, v/v)^31^.

## Additional Information

**How to cite this article**: Li, B. *et al.* Highly efficient removal of lead and cadmium during wastewater irrigation using a polyethylenimine-grafted gelatin sponge. *Sci. Rep.* **6**, 33573; doi: 10.1038/srep33573 (2016).

## Supplementary Material

Supplementary Information

## Figures and Tables

**Table 1:** The isotherm constants of for PEI-grafted gelatin sponge for the adsorption of heavy metals.

Test metal ions	Langmuir model				Freundlich model		
	Q_max_ (mg g^−1^)	K_L_ (L mg^−1^)	R_L_	R^2^	K_F_ (mg g^−1^)	n	R^2^
Pb(II)	80.6	0.0404	0.024	0.9932	6.11	1.27	0.9716
Cd(II)	79.9	0.0404	0.024	0.9896	6.05	1.28	0.9763

**Table 2:** The kinetic constants of PEI-grafted gelatin sponge for the adsorption of heavy metals.

Test metal ions	Pseudo-Frist order			Pseudo-Second order			Intra-particle diffusion model		
	k_1_ (min^−1^)	Q_e,calc*_ (mg g^−1^)	R^2^	k_2_ (g mg^−1^ min^−1^)	Q_e,calc*_ (mg g^−1^)	R^2^	k_i_ (mg g^−1^ min^−1/2^)	C	R^2^
Pb(II)	0.023	57.90	0.9695	0.00082	64.52	0.9968	2.0578	25.739	0.7674
Cd(II)	0.018	50.32	0.9854	0.00073	63.70	0.9964	2.0848	24.059	0.7674

Notes: ^*^Q_e_ denotes the adsorbed amount of heavy metals at the equilibrium concentration and Q_e, calc_ is the calculated value of Q_e_ according to the kinetic model. The experimental values of Q_e_ of Pb(II) and Cd(II) are 61.31 mg g^−1^ and 60.46 mg g^−1^, respectively.
