# Supplementary material for: Highly efficient removal of lead and cadmium during wastewater irrigation using a polyethylenimine-grafted gelatin sponge
Source: Sci Rep. 2016 Sep 16;6:33573. doi: 10.1038/srep33573 (PMC5025662; doi:10.1038/srep33573)
Supplement: Supplementary Information [file srep33573-s1.pdf]

Highly efficient removal of lead and cadmium during wastewater irrigation using a polyethylenimine-grafted gelatin sponge

Bingbing Li[1], Feng Zhou[1], Kai Huang[1], Yipei Wang[2], Surong Mei[1], Yikai Zhou[1], Tao Jing[1*]

[1] State Key Laboratory of Environment Health (Incubation), Key Laboratory of Environment and Health, Ministry of Education, Key Laboratory of Environment and Health (Wuhan), Ministry of Environmental Protection, School of Public Health, Tongji Medical College, Huazhong University of Science and Technology, Wuhan, Hubei, 430030, China

[2] Institute of Environmental Pollution and Health, School of Environmental and Chemical Engineering, Shanghai University, Shanghai 200444, China.

*** Corresponding Author:**

Tao Jing, E-mail: jingtao@hust.edu.cn

Address: School of Public Health, Tongji Medical College, Huazhong University of Science and Technology, #13 Hangkong Road, Wuhan, Hubei, 430030, China

Tel: +86(27)-83552611

Fax: +86(27)-83657765

1. Adsorption isotherms of the PEI-grafting gelatin sponge

The Langmuir isotherm and Freundlich isotherm are widely applied to study equilibrium data of adsorption, which were expressed by the following equations, respectively:

$$Q_e = \frac{K_L Q_{\max} C_e}{1 + K_L C_e} \qquad \text{(Eq. 1)}$$

$$Q_e = K_F C_e^{1/n} \qquad \text{(Eq. 2)}$$

where Q_e is the adsorbed amount of heavy metals at equilibrium concentration (mg g^{-1}), C_e is the equilibrium concentration in the solution (mg L^{-1}), Q_{max} is the maximum adsorption capacity (mg g^{-1}), K_L is the Langmuir adsorption constant (L mg^{-1}), K_F is the Freundlich constant (L g^{-1}), and 1/n is the heterogeneity factor.

The essential characteristics of the Langmuir model can be expressed by a dimensionless constant called the equilibrium parameter (R_L), which is defined as:

$$R_L = \frac{1}{1 + C_{\max} K_L} \qquad \text{(Eq. 3)}$$

where C_{max} is the maximal initial concentration of heavy metals (mg L^{-1}). There are four probabilities for the RL value: (1) $0 < R_L < 1.0$, favorable adsorption; (2) $R_L > 1.0$, unfavorable adsorption; (3) $R_L = 1.0$, linear adsorption; (4) $R_L = 0$, irreversible adsorption.

2. Adsorption kinetic studies of PEI-grafting gelatin sponge

In order to investigate the mechanism and rate-controlling step in the overall adsorption process, pseudo-first order, pseudo-second order and intra-particle diffusion were adopted for a better understanding of the dynamics of heavy metal ions

adsorption on PEI-grafting gelatin sponge. The pseudo-first order and pseudo-second order kinetic models are given as follows:

$$\ln(Q_e - Q_t) = \ln Q_e - k_1 t \qquad \text{(Eq. 4)}$$

$$\frac{t}{Q_t} = \frac{1}{k_2 Q_e^2} + \frac{t}{Q_e} \qquad \text{(Eq. 5)}$$

where Q_e (mg g^{-1}) and Q_t (mg g^{-1}) are the amounts of heavy metals adsorbed at equilibrium and at time t, respectively. k_1 is the pseudo-first order rate constant (min^{-1}), and k_2 is the pseudo-second order rate constant of adsorption (g mg^{-1} min^{-1}). The kinetic parameters in both two models are determined from the linear plots of $\ln(Q_e-Q_t)$ versus t for pseudo-first order model and t/Q_t versus t for pseudo-second order model. The validity of each model was checked by the fitness of the straight line (R^2) as well as the experimental and calculated values of Q_e.

The two models above cannot identify the diffusion mechanism during the adsorption process, so the experimental data are tested by the intra-particle diffusion model, which can be expressed by following equation:

$$Q_t = k_i t^{1/2} + C \qquad \text{(Eq. 6)}$$

where k_i is the intra-particle diffusion rate constant (mg g^{-1} $min^{-1/2}$) and C is the intercept. The value of C (mg g^{-1}) and k_i can be obtained from the intercept and slope of the linear plot of Q_t versus $t^{1/2}$, respectively.

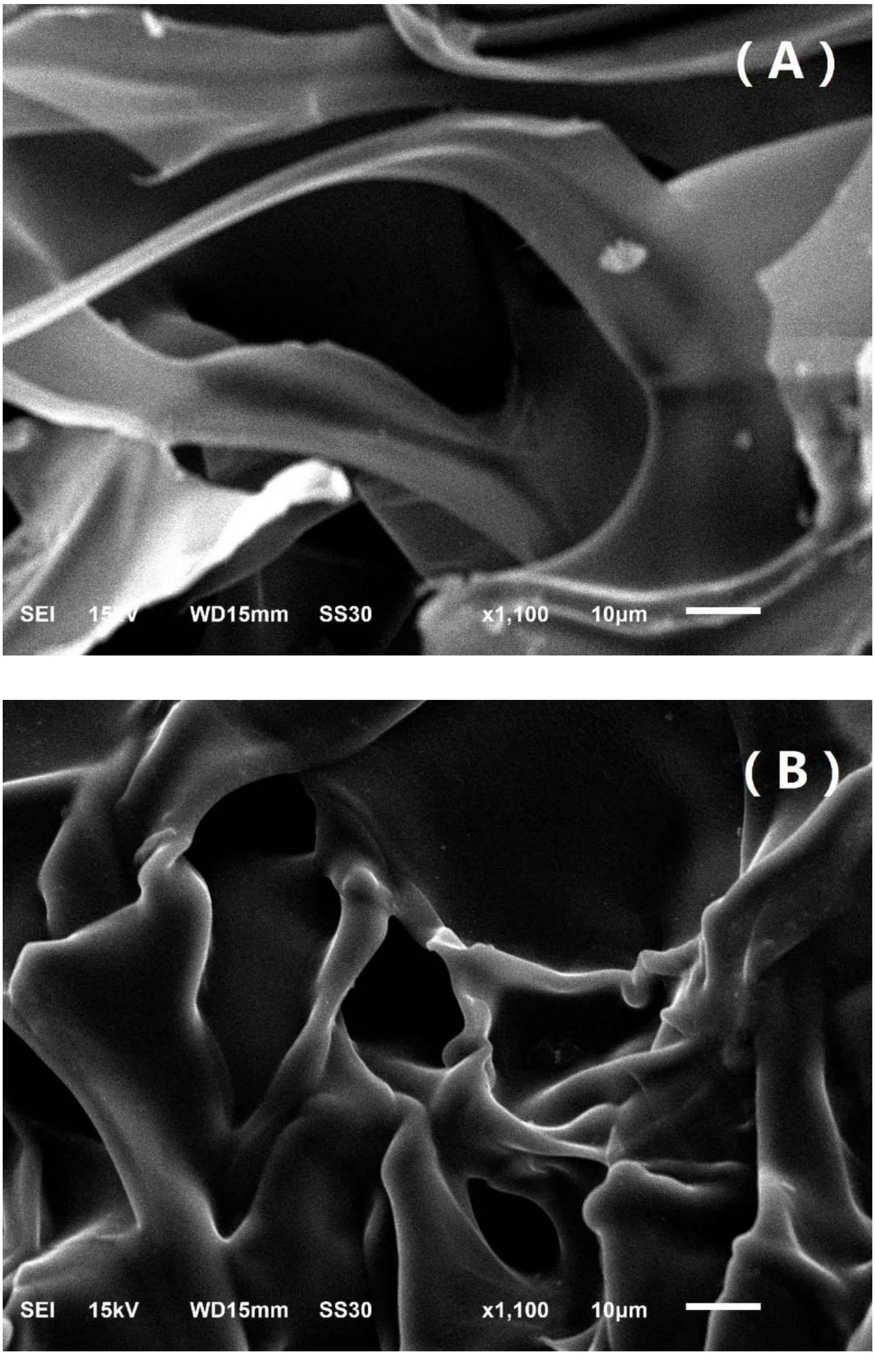

Figure S1. SEM micrographs of the gelatin sponge (A) and PEI-grafted gelatin sponge (B).

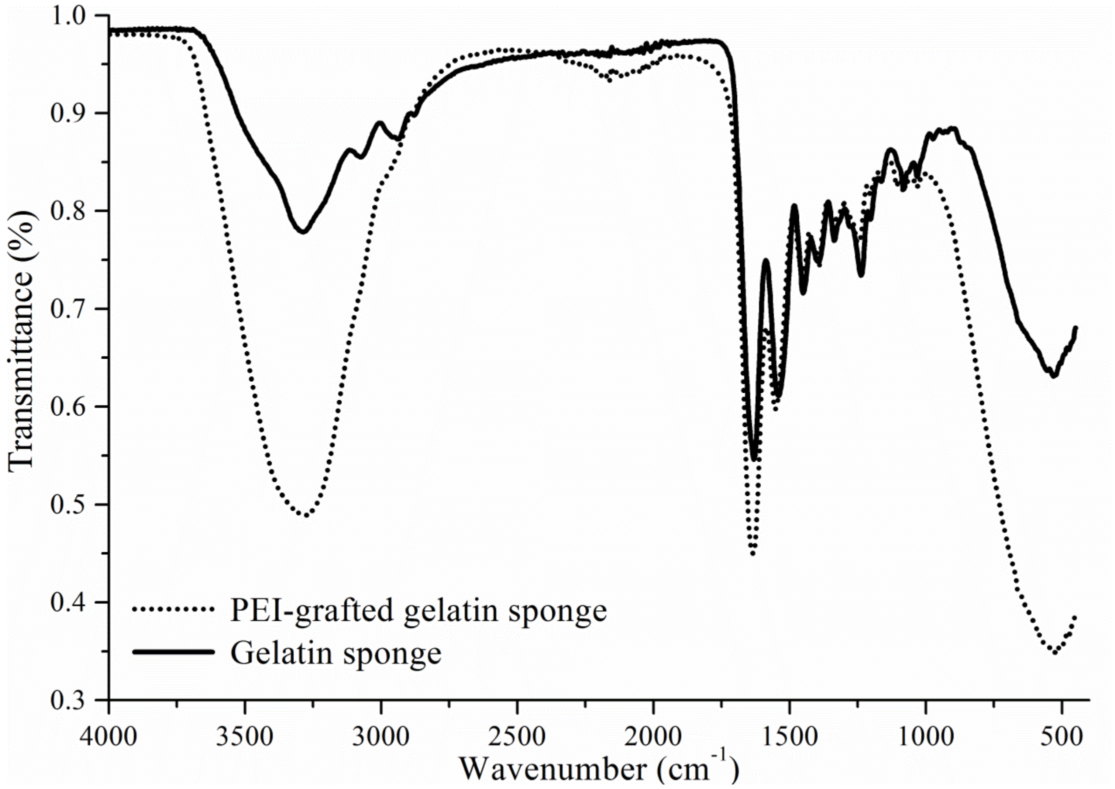

Figure S2. FTIR spectra of the gelatin sponge and the PEI-grafted gelatin sponge.

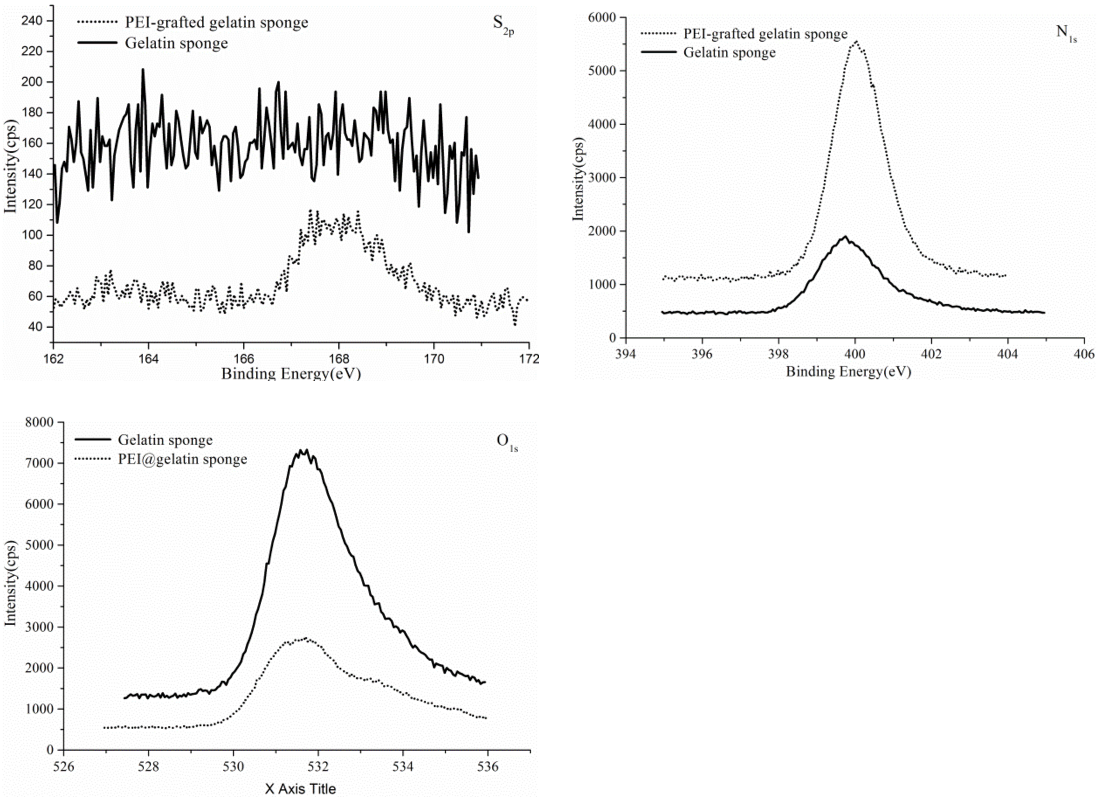

Figure S3. XPS spectrum of the gelatin sponge and the PEI-grafted gelatin sponge.

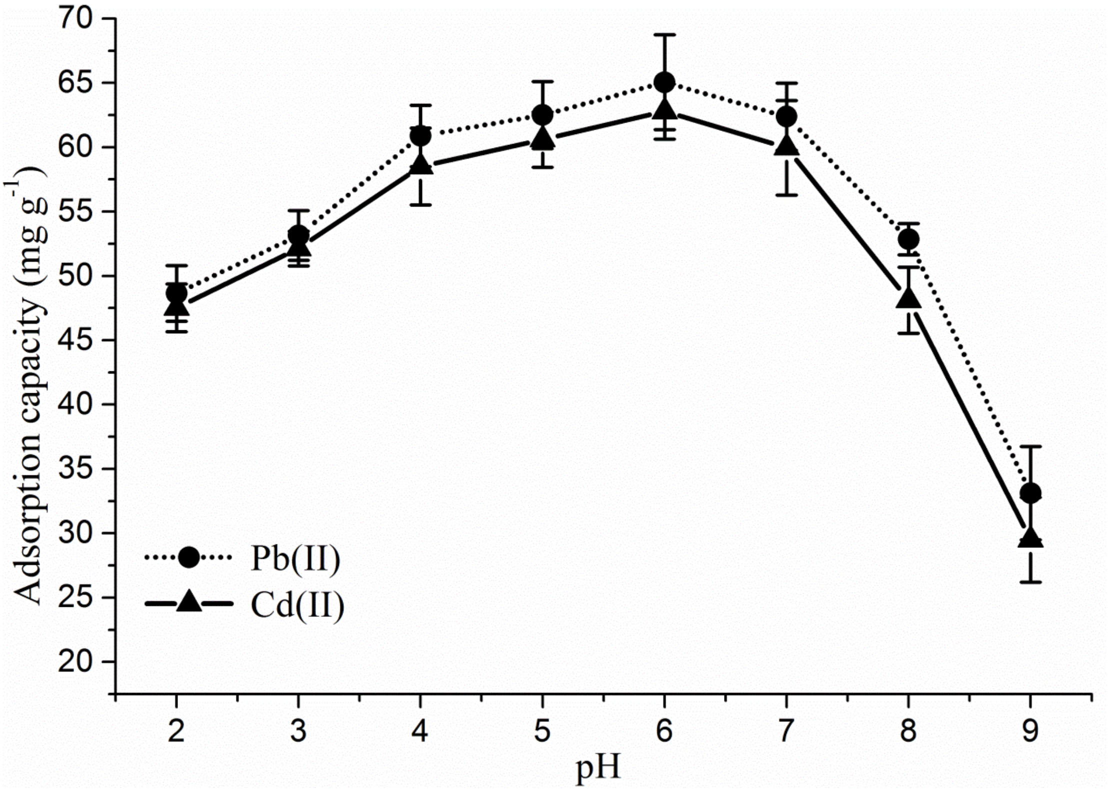

Figure S4. Effect of pH on the removal efficiency of heavy metals.

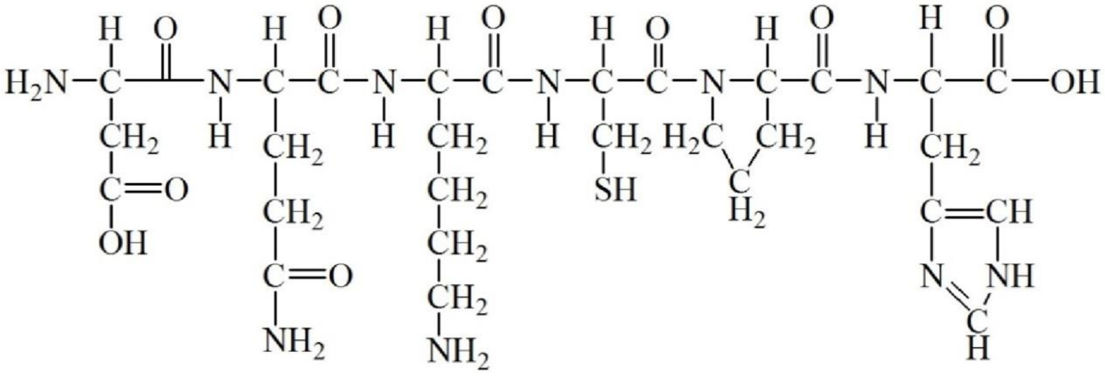

Figure S5. A typical structure of gelatin polypeptide.

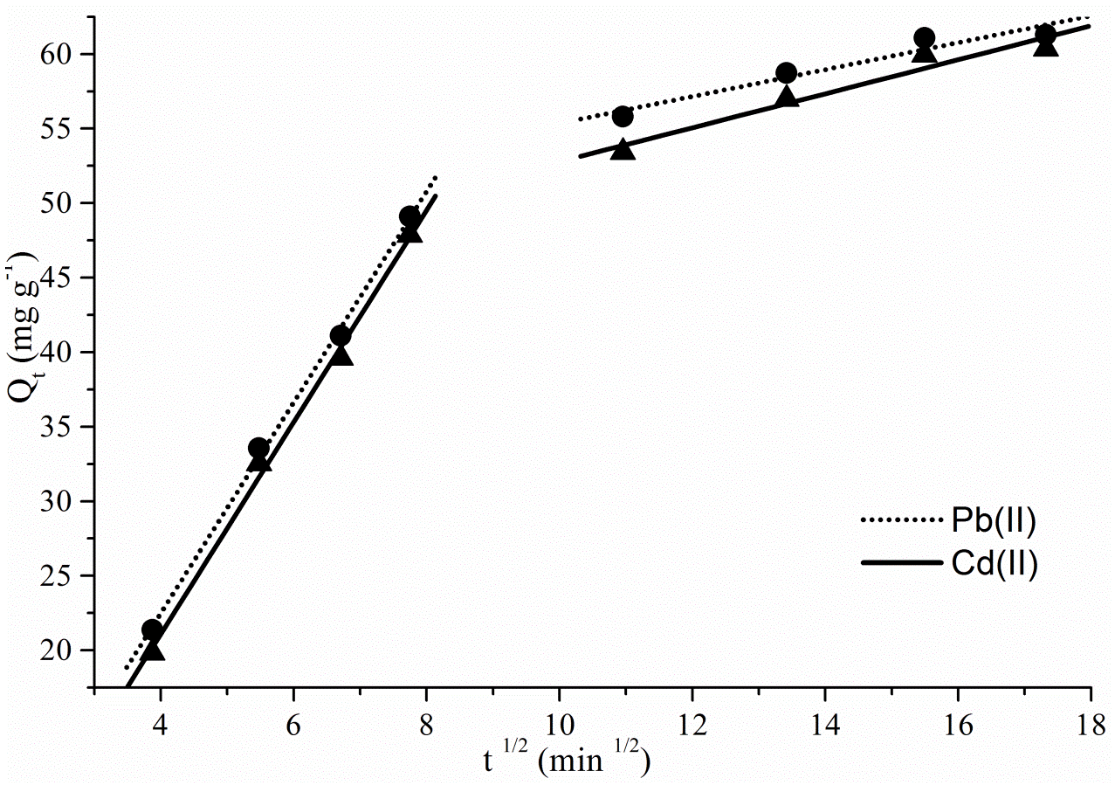

Figure S6. Intraparticle diffusion treatment of Pb(II) and Cd(II) on the PEI-graftedgelatin sponge.
